# Contemporary reliance on bicarbonate acquisition predicts increased growth of seagrass *Amphibolis antarctica* in a high-CO_2_ world

**DOI:** 10.1093/conphys/cou052

**Published:** 2014-11-27

**Authors:** Owen W. Burnell, Sean D. Connell, Andrew D. Irving, Jennifer R. Watling, Bayden D. Russell

**Affiliations:** 1 School of Earth & Environmental Sciences, University of Adelaide, Adelaide, SA 5005, Australia; 2 School of Medical and Applied Sciences, Central Queensland University, Bruce Highway, Rockhampton, QLD 4702, Australia

**Keywords:** *Amphibolis antarctica*, carbon dioxide, carbonic anhydrase, electron transport rate, oxygen evolution, photosynthesis

## Abstract

Rising atmospheric CO_2_ is increasing the availability of dissolved CO_2_ in the ocean relative to HCO_3_^−^. Currently, many marine primary producers use HCO_3_^−^ for photosynthesis, but this is energetically costly. Increasing passive CO_2_ uptake relative to HCO_3_^−^ pathways could provide energy savings, leading to increased productivity and growth of marine plants. Inorganic carbon-uptake mechanisms in the seagrass *Amphibolis antarctica* were determined using the carbonic anhydrase inhibitor acetazolamide (AZ) and the buffer tris(hydroxymethyl)aminomethane (TRIS). *Amphibolis antarctica* seedlings were also maintained in current and forecasted CO_2_ concentrations to measure their physiology and growth. Photosynthesis of *A. antarctica* was significantly reduced by AZ and TRIS, indicating utilization of HCO_3_^−^-uptake mechanisms. When acclimated plants were switched between CO_2_ treatments, the photosynthetic rate was dependent on measurement conditions but not growth conditions, indicating a dynamic response to changes in dissolved CO_2_ concentration, rather than lasting effects of acclimation. At forecast CO_2_ concentrations, seedlings had a greater maximum electron transport rate (1.4-fold), photosynthesis (2.1-fold), below-ground biomass (1.7-fold) and increase in leaf number (2-fold) relative to plants in the current CO_2_ concentration. The greater increase in photosynthesis (measured as O_2_ production) compared with the electron transport rate at forecasted CO_2_ concentration suggests that photosynthetic efficiency increased, possibly due to a decrease in photorespiration. Thus, it appears that the photosynthesis and growth of seagrasses reliant on energetically costly HCO_3_^−^ acquisition, such as *A. antarctica*, might increase at forecasted CO_2_ concentrations. Greater growth might enhance the future prosperity and rehabilitation of these important habitat-forming plants, which have experienced declines of global significance.

## Abstract (precis)

We find energetically costly bicarbonate pathways exist in three temperate seagrasses and then provide evidence that indicates greater growth and photosynthetic efficiency for bicarbonate users in a high CO2 world. Greater growth might enhance the future prosperity and rehabilitation of these important habitat forming plants, which have experienced declines of global significance.

## Introduction

Seagrasses are habitat-forming marine plants that provide a number of critical ecological services to coastal zones, such as stabilization of sediments, support of trophic food webs, nutrient cycling and carbon sequestration (Costanza *et al.*, 1997; Duarte, 2002; Waycott *et al.*, 2009; Irving *et al.*, 2011). When angiosperms first entered the aquatic realm nearly 90 million years ago, atmospheric CO_2_ levels were much greater (approximately three to seven times) than today (Beer and Koch, 1996; Beardall *et al.*, 1998; Berner and Kothavala, 2001). Since that time, atmospheric CO_2_ concentrations have generally declined (Berner and Kothavala, 2001), a trend which has resulted in potential carbon limitation for many marine plants. This low atmospheric CO_2_ has reduced the availability of inorganic carbon (C_i_) for photosynthesis, which is compounded in marine systems by the slow diffusion of CO_2_ in seawater and the slow rate of conversion of HCO_3_^−^ to CO_2_ when uncatalysed (Beer, 1989; Larkum *et al.*, 1989; Schwarz *et al.*, 2000). The recent spike in atmospheric CO_2_ linked to anthropogenic activities is changing C_i_ availability in marine systems and is thus likely to affect carbon acquisition and growth in primary producers, such as seagrasses (Koch *et al.*, 2013).

While HCO_3_^−^ is more readily available than dissolved CO_2_ in marine systems, it cannot diffuse passively across the cell plasma membrane, and therefore, extracellular mechanisms have evolved to aid aquatic plants in the acquisition of CO_2_ from HCO_3_^−^ for photosynthesis, thereby reducing C_i_ limitation (Larkum, *et al.*, 1989; Invers *et al.*, 1999; Hellblom *et al.*, 2001). Three primary extracellular HCO_3_^−^ acquisition systems have been described for seagrasses (Beer and Koch, 1996; James and Larkum, 1996; Hellblom *et al.*, 2001; Beer *et al.*, 2002). First, the enzyme carbonic anhydrase (CA) can catalyse the rapid conversion of HCO_3_^−^ to CO_2_ to restore CO_2_/HCO_3_^−^ equilibrium at the plasma membrane or concentrate CO_2_ at the chloroplast level (System A, *sensu* Beer *et al.*, 2002). Second, the outward pumping of protons (H^+^) from cells can create H^+^ gradients to aid the cotransport of H^+^ and HCO_3_^−^ back across the plasma membrane (System B). Finally, the combination of extracellular CA-catalysed dehydration of HCO_3_^−^ to CO_2_ within acidified zones created by the extrusion of H^+^ across the plasma membrane can concentrate CO_2_ and encourage diffusion into cells (System C). For a full review of these mechanisms, see Beer *et al.* (2002).

All seagrasses tested to date appear to be reliant, to some extent, on the extracellular activity of CA for carbon acquisition, suggesting that they could experience some degree of carbon limitation at current atmospheric CO_2_ concentrations (Larkum *et al.*, 2006; Koch *et al.*, 2013). Also, there is an energetic cost associated with both the production of extracellular CA and the more active mode of carbon acquisition using H^+^ extrusion (Spalding and Ogren, 1982; Raven and Lucas, 1985; Falk and Palmqvist, 1992; Kübler and Raven, 1995; Fridlyand *et al.*, 1996). As atmospheric, and thus oceanic, CO_2_ continues to accumulate, this energetic cost could diminish if reliance on these carbon-acquisition mechanisms decreases, relative to direct CO_2_ usage (Beardall and Giordano, 2002; Raven *et al.*, 2011; Koch *et al.*, 2013). For example, some lower order marine producers (i.e. cyanobacteria and eukaryotic algae) can adjust their HCO_3_^−^-acquisition strategies within a number of hours of exposure to different CO_2_ conditions (Falk and Palmqvist, 1992; Matsuda and Colman, 1995; Brueggeman *et al.*, 2012). Seagrasses may have a similar ability to regulate energetically costly C_i_ acquisition, but our understanding of this has advanced slowly relative to lower order producers (i.e. cyanobacteria and eukaryotic algae) that have shorter generation times and can therefore be manipulated more easily for genetic expression studies (Larkum, *et al.*, 2006).

Increasing CO_2_ availability and any down-regulation of HCO_3_^−^ acquisition could result in improved photosynthetic efficiency as the energy required to acquire carbon decreases, which may also translate to greater photosynthetic rates (Badger and Andrews, 1982; Raven *et al.*, 2011; Koch *et al.*, 2013). Likewise, if CA-mediated HCO_3_^−^ mechanisms are maintained, they may become more efficient at lower ambient pH levels (Koch *et al.*, 2013). However, this potential for more efficient and greater photosynthesis may be accompanied by a net gain in leaf growth or energy storage only when other resources, such as light or nitrogen, are not limiting (Zimmerman *et al.*, 1997; Palacios and Zimmerman, 2007; Alexandre *et al.*, 2012). In many seagrass species with a heavy reliance on HCO_3_^−^ for C_i_ acquisition, it is unknown whether they will undergo subsequent changes in growth as dissolved CO_2_ increases.

In the present study, the reliance of *Amphibolis antarctica* (Labill.) Sonder et Ascherson on HCO_3_^−^ pathways of C_i_ acquisition was investigated by using an inhibitor of the enzyme CA (i.e. acetazolamide, AZ) and the biological buffer (i.e. tris(hydroxymethyl)aminomethane, TRIS). Having established that *A. antarctica* has a significant reliance on energetically costly HCO_3_^−^ acquisition, a second experiment was conducted in which juvenile *A. antarctica* were grown in the presence of ambient (∼390 ppm) and forecasted CO_2_ concentrations (∼900 ppm). It was hypothesized that photosynthesis and growth would increase for *A. antarctica* when CO_2_ was enriched, because the greater availability of CO_2_ relative to HCO_3_^−^ might increase the photosynthetic efficiency of plants, as they may partition relatively fewer resources to energetically costly processes, such as HCO_3_^−^-uptake mechanisms or photorespiration.

## Materials and methods

### Plant material

Mature seagrasses were collected from a depth of 4 m at Marino Rocks in the Gulf St Vincent, South Australia (35°02.806 S, 138°30.350 E). Seagrasses were transported to The University of Adelaide and kept in recirculating aerated aquaria with lighting conditions similar to the collection site (∼60 µmol m^−2^ s^−1^) in a 12 h–12 h light–dark cycle for 1 week, during which time experiments to determine C_i_-uptake mechanisms took place.

### Inorganic carbon-acquisition mechanisms

The C_i_-uptake mechanisms of seagrasses were investigated by inhibiting HCO_3_^−^ pathways to carbon acquisition. Seagrasses were exposed to the inhibitor AZ or the biological buffer TRIS, either separately or in combination. The primary seagrass of interest was *A. antarctica*, but to identify the generality of the mechanism within and across genera, C_i_-uptake mechanisms were also investigated for two other co-occurring seagrass species, the congener *Amphibolis griffithii* (Black) den Hartog and the species *Posidonia sinuosa* (Cambridge and Kuo), using the same methodology.

Oxygen evolution rates were determined using a Clark-type oxygen electrode and the logging program Biograph (Axword Software, Adelaide, South Australia). An entire leaf of *A. antarctica* (∼20 mm long) that was free of epiphyte growth was placed in the electrode chamber in 4 ml of seawater filtered to 0.45 µm. The fourth or fifth youngest leaf on each leaf head was chosen because these leaves were mature and consistently free of epiphytes and other biota, as well as being the correct length to fit the photosynthetic chamber. The chamber was illuminated using a fibre-optic light, which delivered a photon flux density (PFD) of ∼500 µmol m^−2^ s^−1^ at the leaf surface that was sufficient to saturate photosynthesis in *A. antarctica*. This light level was chosen to represent peak irradiances previously recorded in *A. antarctica* meadows (A. D. Irving, unpublished data; Bryars *et al.*, 2011), but below levels shown to have a photoinhibitory effect on *A. antarctica* and other co-occurring species (Masini *et al.*, 1995; Ralph *et al.*, 1998). Light between 380 and 710 nm was measured by positioning the fibre quantum sensor of a diving pulse amplitude modulated (PAM) fluorometer (Walz, Effeltrich, Germany) in the electrode chamber. The PAM fluorometer fibre quantum sensor was calibrated against a LI-COR quantum sensor (Li-192SA; Lincoln, NE, USA). The electrode chamber was maintained at a constant temperature of 20°C using a transparent recirculating water jacket and was constantly mixed using a magnetic stirrer.

Seagrass leaves were sealed in the chamber and covered with a black cloth to measure dark respiration. Respiration rates were allowed to stabilize for 3 min before recording. The chamber was then illuminated, and where necessary, the appropriate stock solutions (see below) were injected into the chamber using micro-syringes. Photosynthetic rates were allowed to stabilize for 2 min before recording. Photosynthetic and respiration rates were averaged over 3 min and are expressed on a chlorophyll basis (as micromoles of oxygen per gram of chlorophyll per minute).

A 20 mm stock solution of the CA inhibitor AZ (Sigma Aldrich) was prepared in 50 mm sodium hydroxide (NaOH). A 1 m stock solution of TRIS (Sigma Aldrich) was prepared and the pH adjusted to ambient seawater pH (8.05). The buffer yielded a pH of 8.06 when injected into the electrode chamber. For AZ, 20 µl of stock solution was injected to achieve a final chamber concentration of 100 µm. For TRIS, 200 µl of stock solution was injected to achieve a final chamber concentration of 50 mm. Chamber conditions, including carbonate chemistry, are presented in Table 1a.

**Table 1:** Sea water chemistry in the oxygen electrode chamber during buffer/inhibitor experiments (**a**; see Figs 1 and S1), during 12 week growth experiments (**b**; see Figs 2a and 3) and reciprocal switch measurements (**c**; Fig. 2b)

	pH	**A**_T_ [µmol (kg sea water^−1^)]	Salinity (‰)	Temperature (°C)	Total CO_2_ [µmol (kg sea water^−1^)]	Partial pressure of CO_2_ (µatm)	HCO_3_^−^ [µmol (kg sea water^−1^)]	CO_3_ [µmol (kg sea water^−1^)]	CO_2_ [µmol (kg sea water^−1^)]
(a)									
Control	8.05 ± 0.01	2533 ± 49	38	20	2223	440	1981	228	14.0
TRIS	8.06 ± 0.01	2533 ± 49	38	20	2215	424	1968	234	13.5
AZ	8.08 ± 0.02	2533 ± 49	38	20	2204	404	1950	241	12.9
TRIS + AZ	8.06 ± 0.00	2533 ± 49	38	20	2211	418	1962	236	13.3
(b)									
L[CO_2_]	8.12 ± 0.002	2701 ± 27	40.3 ± 0.06	19.8 ± 0.01	2309	376	2011	285	11.8
H[CO_2_]	7.82 ± 0.003	2697 ± 35	40.0 ± 0.05	19.9 ± 0.01	2498	872	2309	161	27.5
(c)									
L[CO_2_]	8.15 ± 0.003	2648 ± 43	40.6 ± 1.49	20	2236	338	1930	295	10.6
H[CO_2_]	7.82 ± 0.007	2656 ± 48	40.3 ± 1.35	20	2454	849	2266	162	26.7

Abbreviations: AZ, acetazolamide; TRIS, tris(hydroxymethyl)aminomethane. L[CO_2_] = low CO_2_, H[CO_2_] = high CO_2_. Measurements of pH, total alkalinity (**A**_T_), salinity and temperature (fixed at 20°C during photosynthetic trials) were used to calculate carbonate chemistry. Salinity was fixed at 38‰ during inhibitor experiments.

### Effects of CO_2_ on photosynthesis and growth

Juvenile *A. antarctica* seedlings were collected at the same depth and location as described above for adult plants and transported to an outdoor glasshouse at The University of Adelaide. Four individual seedlings were planted in sediment from the collection site in each of 12 transparent 2 litre microcosms (25 cm depth). Seagrasses were maintained at two CO_2_ concentrations, representing current atmospheric CO_2_ levels (∼390 ppm) and forecasted future CO_2_ levels (∼900 ppm) under emission scenario A1FI for the year 2100 (Meehl *et al.*, 2007), hereafter referred to as low CO_2_ (L[CO_2_]) and high CO_2_ (H[CO_2_]), respectively. Carbon dioxide was enriched by aerating the microcosms with a combination of ambient air and pure CO_2_ using a two-channel gas mixer (Columbus Instruments, Columbus, OH, USA), which is equivalent to maintaining treatments within an enriched CO_2_ atmosphere. Aeration helps to accelerate the diffusion of CO_2_ into seawater, also minimizing any variation driven by plant photosynthesis or respiration. The accuracy of the mixed gas aerating treatments can vary slightly dependent on ambient atmospheric conditions, but any such changes are minor and occur naturally whenever ambient air is used for aeration; ambient CO_2_ treatments are also subject to such natural fluctuations. Experimental conditions, including carbonate chemistry, in experimental microcosms were monitored throughout the experiment (Table 1b). Seventy per cent of seawater was replaced twice weekly to maintain salinity and alkalinity.

Microcosms were in an outdoor glass house and they were shaded from full surface irradiance using a combination of 50 and 70% shade cloth. HOBO^®^ waterproof light loggers (Onset, Wareham, MA, USA) were used to record light in lux, which was then converted to give an approximation of photosynthetically active radiation using the constant for natural sunlight (1 lux = 54 µmol m^−2^ s^−1^) recommended by Thimijan and Heins (1983). Average daily PFD was 42.5 µmol m^−2^ s^−1^ over a 12 h photoperiod, with an average daily maximum of 111.3 ± ’11.7 µmol m^−2^ s^−1^ recorded at 13.00 h. Shading was designed to replicate closely the light conditions recorded during a monitoring study on the Adelaide metropolitan coast, which found average daily PFD of ∼44 µmol m^−2^ s^−1^ over a 12 h photoperiod (Irving, 2009). One specific 40 day light monitoring deployment at ∼2 m (low tide) on the Adelaide metropolitan coast found average daily maximal PFD of 155.95 ± 20.54 µmol m^−2^ s^−1^ (A. D. Irving, unpublished data), which is comparable to the peak levels recorded in our acclimation study. The daily maximum in this monitoring study ranged from 7 to 453 µmol m^−2^ s^−1^, highlighting the dynamic nature of the light climate in the shallow sub-tidal near-shore zone, where the swell direction, wind direction, rainfall, runoff and topography can all influence turbidity and thus light attenuation.

### Chlorophyll fluorescence

A submersible diving PAM fluorometer (Walz, Effeltrich, Germany) was used to record rapid light curves (RLCs) and maximal quantum yield (QY_max_). Seagrasses were measured *in situ* in microcosms at L[CO_2_] and H[CO_2_] after 12 weeks. The photon flux densities used during the RLCs were 0, 18, 37, 62, 92, 125, 186, 256 and 400 µmol m^−2^ s^−1^, each of 10 s duration, followed by a saturating pulse of light, to record the effective quantum yield (Φ_PSII_). All RLC measurements were taken between 11.00 and 12.30 h. The light absorbance of every sample leaf was measured by placing a quantum sensor directly behind the leaf and recording the percentage of ambient light that was absorbed. The electron transport rate (ETR) was calculated as follows: ETR = Φ_PSII_ × PFD × leaf absorbance × 0.5. For quantitative comparisons of RLCs [i.e. initial slope (α), maximal electron transport rate (ETR_max_) and light saturation (*I*_k_)], the ETR data were fitted with a least-squares non-linear regression curve based on the exponential difference equation from Platt *et al.* (1980) using the Microsoft Excel (Microsoft, Redmond, WA, USA) solvers provided by Ritchie (2008). Light saturation was calculated as ETR_max_ /α, where α is the initial slope of the non-inhibited section of the fitted curve. The value of QY_max_ was determined from pre-dawn fluorescence measurements.

### Photosynthesis and respiration

Following 12 weeks at experimental CO_2_ levels, photosynthesis and respiration rates were measured as described above, but without the addition of buffers and inhibitors. Reciprocal measurements were made, where individuals from each CO_2_ treatment were measured at both low and high CO_2_, to determine whether physiological acclimation had occurred over the course of the experiment (see Table 1c for a description of chamber conditions). Electron transport rates at 400 µmol m^−2^ s^−1^ and maximal gross photosynthetic rates were then used to calculate ETR-to-O_2_ ratios (i.e. moles of electrons per mole of O_2_ evolved). This was done for L[CO_2_] and H[CO_2_] plants tested in their respective growth treatments.

### Growth

Growth parameters were measured after 12 weeks. Leaves and shoots (above ground) were separated from roots and rhizomes (below ground) and dried for 48 h at 60°C to measure dry mass (DM). Above-ground components were then washed in 5% HCl to remove any calcified epiphytes, redried for 48 h at 60°C and reweighed.

Leaf initiation was recorded by trimming the corner of the second youngest leaf on the highest leaf head of each seedling at the commencement of the growth experiment. On juveniles, this method of monitoring leaf growth is less obtrusive than sheath marking or using leaf ties, which can impact meristem growth and plant buoyancy (O. W. Burnell, personal observation). The total number of leaves on each leaf head was recorded before planting and again after 12 weeks to calculate the change in total leaf number, which accounted for different rates of leaf shedding and production from any new meristems.

### Chlorophyll determination

Chlorophyll content was measured to enable expression of photosynthesis and respiration per unit of chlorophyll. Following oxygen electrode measurements, seagrass material was removed from the chamber, rinsed in milliQ water, and immediately frozen in liquid nitrogen. Samples were then stored at −80°C until extractions were conducted. Chlorophyll concentrations were determined using the methodology of Granger and Lizumi (2001). Briefly, leaves were soaked in 1 ml of 100% acetone in dark, refrigerated conditions for 1 h. Seagrass and acetone were then transferred to a chilled mortar and pestle and ground using an additional 1 ml of 86.7% acetone and acid washed sand until they were reduced to a flocculent slurry. Two drops of 1% MgCO_3_ were added before diluting to 4 ml with 86.7% acetone to achieve a final sample concentration of 90% acetone. Samples were then centrifuged for 5 min at 670.8 *g* of relative centrifugal force. Absorbance of the supernatant was measured in a spectrophotometer (Jenway 6405, Stone, Staffordshire, UK) at 647, 664 and 725 nm. Chlorophyll concentrations were calculated using equations from Jeffrey and Humphrey (1975).

## Results

**Figure 1:**
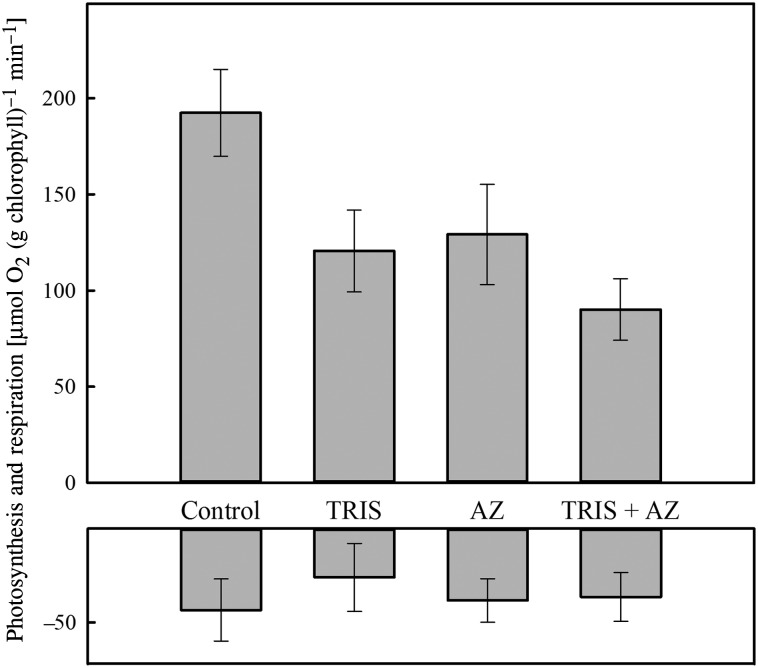
The effects of inhibitors [tris(hydroxymethyl)aminomethane (TRIS), acetazolamide (AZ) and TRIS + AZ] on rates of photosynthesis and respiration in *Amphibolis antarctica* measured in seawater. Bars are means ± SEM (*n* = 8).

It was evident that *A. antarctica* uses HCO_3_^−^ pathways for C_i_ acquisition, because light-saturated photosynthesis declined by ∼54% when AZ and TRIS were added in combination (Fig. 1). The sensitivity of *A. antarctica* to both AZ and the TRIS buffer was consistent with a System A + B mode of C_i_ acquisition (Beer *et al.*, 2002). The lack of an interactive effect in the two-factor ANOVA provided no support for a System C mode of acquisition (Table 2a). Inorganic carbon uptake in the congener *A. griffithii* followed a similar pattern to *A. antarctica*, in that photosynthesis declined by ∼57% when AZ and TRIS were added in combination; however, an interaction between AZ and TRIS in the two-factor ANOVA provided better support for a System C mode of acquisition (*F*_1,28_ = 7.88, *P* = 0.008; Supplementary material Fig. S1a and Supplementary material Table S1). In contrast, the species *P. sinuosa* was insensitive to the addition of the TRIS buffer and only moderately affected by the inhibitor AZ, highlighting a lesser dependency on HCO_3_^−^ pathways for photosynthesis (Supplementary material Fig. S1b and Supplementary material Table S1). Dark respiration did not differ between experimental treatments for any of the three species, and therefore, analyses are not presented (Fig. 1 and Supplementary material Fig. S1). For rates of photosynthesis and respiration per gram of fresh mass, see Supplementary material Table S2.

**Table 2:** ANOVA for the effects on photosynthesis in *A. antarctica* of buffer/inhibitor (**a**; in seawater/control, TRIS, AZ and TRIS + AZ; see Fig. 1) and reciprocal switch (**b**; i.e. plants were grown at either L[CO_2_] or H[CO_2_] and then measured at both L[CO_2_] and H[CO_2_]; see Fig. 2b)

Source	d.f.	MSE	*F*	*P*-Value
(a) Buffer/inhibitor, *A. antarctica*				
TRIS	1	24 540	6.36	0.016
AZ	1	17 517	4.54	0.035
TRIS × AZ	1	2157	0.56	0.467
Residual	28	3858		
(b) Reciprocal switch, *A. antarctica*				
Measurement	1	578 810	9.66	0.005
Growth	1	23 996	0.40	0.574
Measurement × growth	1	90 060	1.50	0.252
Residual	16	59 916		

Abbreviations: d.f., degrees of freedom; *F*, f-statistic; MSE, mean squared error. No significant differences were found between respiration rates; therefore, analyses are not presented.

**Figure 2:**
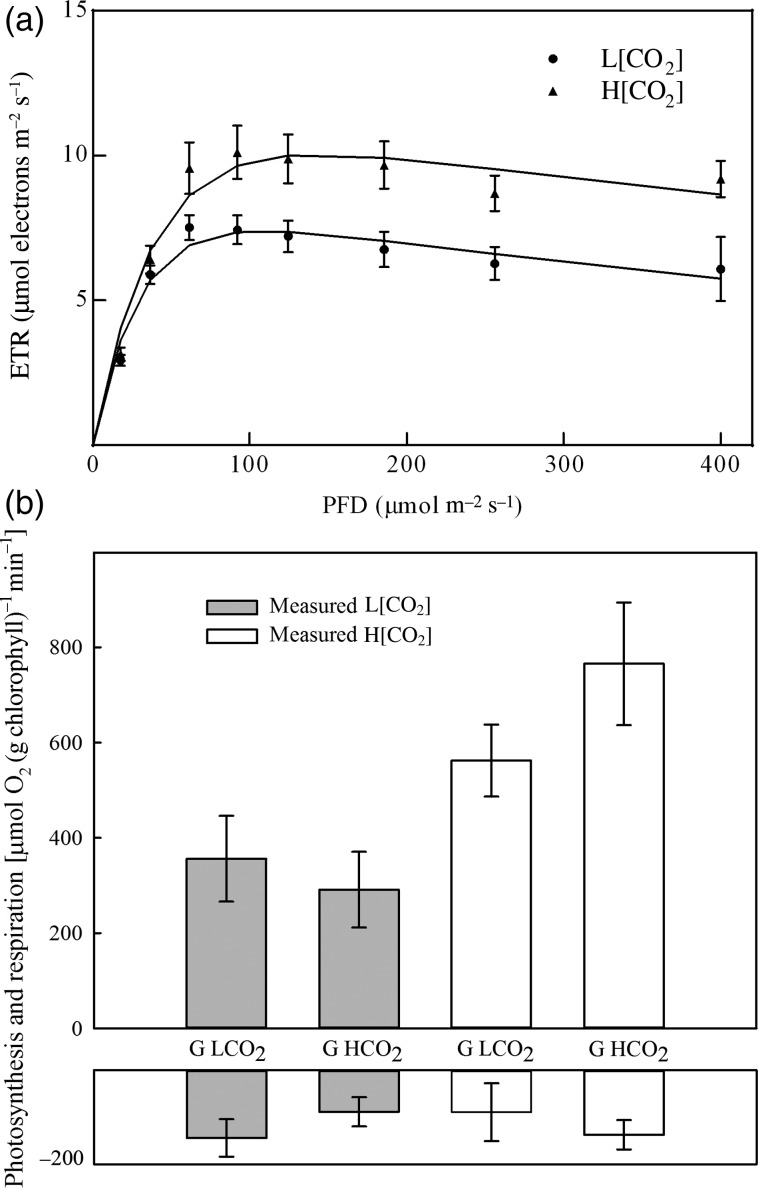
(**a**) Rapid light curves of electron transport rate for *A. antarctica* grown and measured at 390 (L[CO_2_]) or 900 ppm (H[CO_2_]). Filled circles indicate L[CO_2_] and filled triangles H[CO_2_]. Data points are means ± SEM (*n* = 6). (**b**) Rates of photosynthesis and respiration in *A. antarctica* measured at either low (shaded bars) or high CO_2_ (open bars) and grown at either low (G L[CO_2_]) or high CO_2_ (G H[CO_2_]). Bars are means ± SEM (*n* = 5).

Forecasted CO_2_ increased both the ETR and photosynthesis of *A. antarctica* when measured in growth conditions (Fig. 2 and Table 2b). The mean ETR_max_ from the fitted regression curves was 7.39 ± 0.80 µmol electrons m^−2^ s^−1^ for L[CO_2_] plants, compared with 10.04 ± 0.55 µmol electrons m^−2^ s^−1^ for H[CO_2_] plants (*F*_1,10_ = 7.561, *P* = 0.028). The onset of light saturation (*I*_k_) for ETR occurred at 27.12 ± 2.60 µmol m^−2^ s^−1^ for L[CO_2_], compared with 35.79 ± 2.82 µmol m^−2^ s^−1^ for H[CO_2_] plants (*F*_1,10_ = 5.13, *P* = 0.049). In contrast, there was no difference in the initial slope (α) of ETR (0.28 ± 0.03 and 0.29 ± 0.02) or QY_max_ (0.656 ± 0.033 and 0.683 ± 0.017) for L[CO_2_] and H[CO_2_], respectively, thus analyses are not presented.

When plants were reciprocally switched between CO_2_ treatments, measurement CO_2_ had a significant effect on photosynthesis of both L[CO_2_] and H[CO_2_] grown plants (Fig. 2b and Table 2b). In contrast, there was no effect of growth CO_2_; that is, seagrasses photosynthesis was responding primarily to measurement conditions (i.e. dissolved CO_2_), rather than exhibiting significant physiological acclimation to growth in the different CO_2_ treatments. Molar ratios between ETR and gross oxygen evolution based on leaf area were 9.51 mol electrons (mol O_2_)^−1^ for L[CO_2_] plants and 8.08 mol electrons (mol O_2_)^−1^ for H[CO_2_] plants.

**Figure 3:**
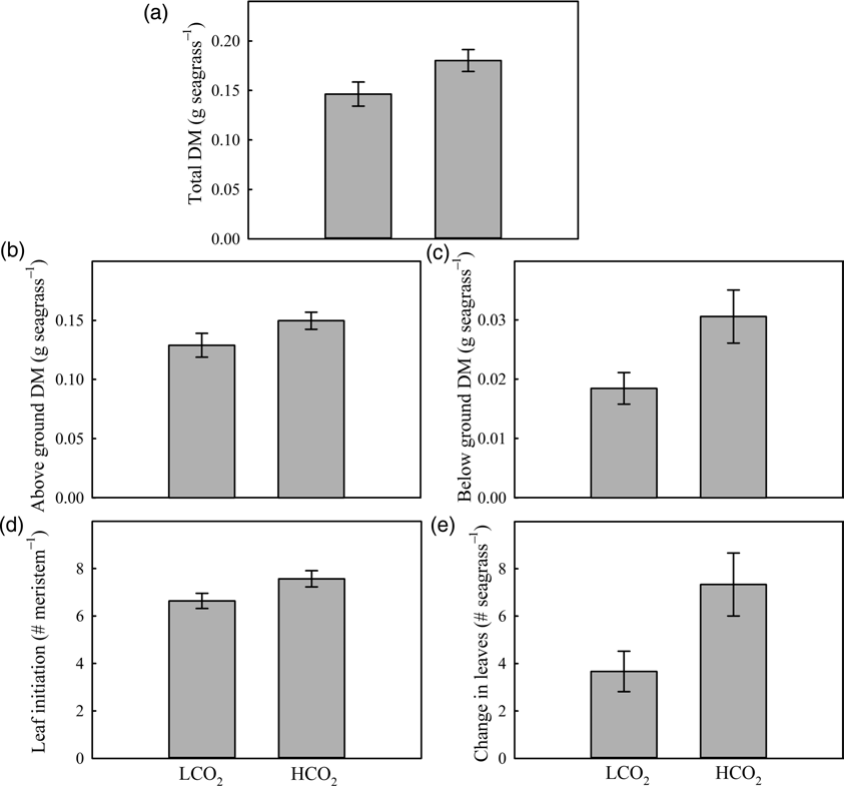
Effect of growth [CO_2_] on total dry mass (DM; **a**), above-ground DM (**b**), below-ground DM (**c**), leaf initiation (**d**) and change in leaves per plant (**e**) for *A. antarctica* grown in L[CO_2_] and H[CO_2_] conditions for 12 weeks. Bars are means ± SEM (*n* = 6).

**Figure 4:**
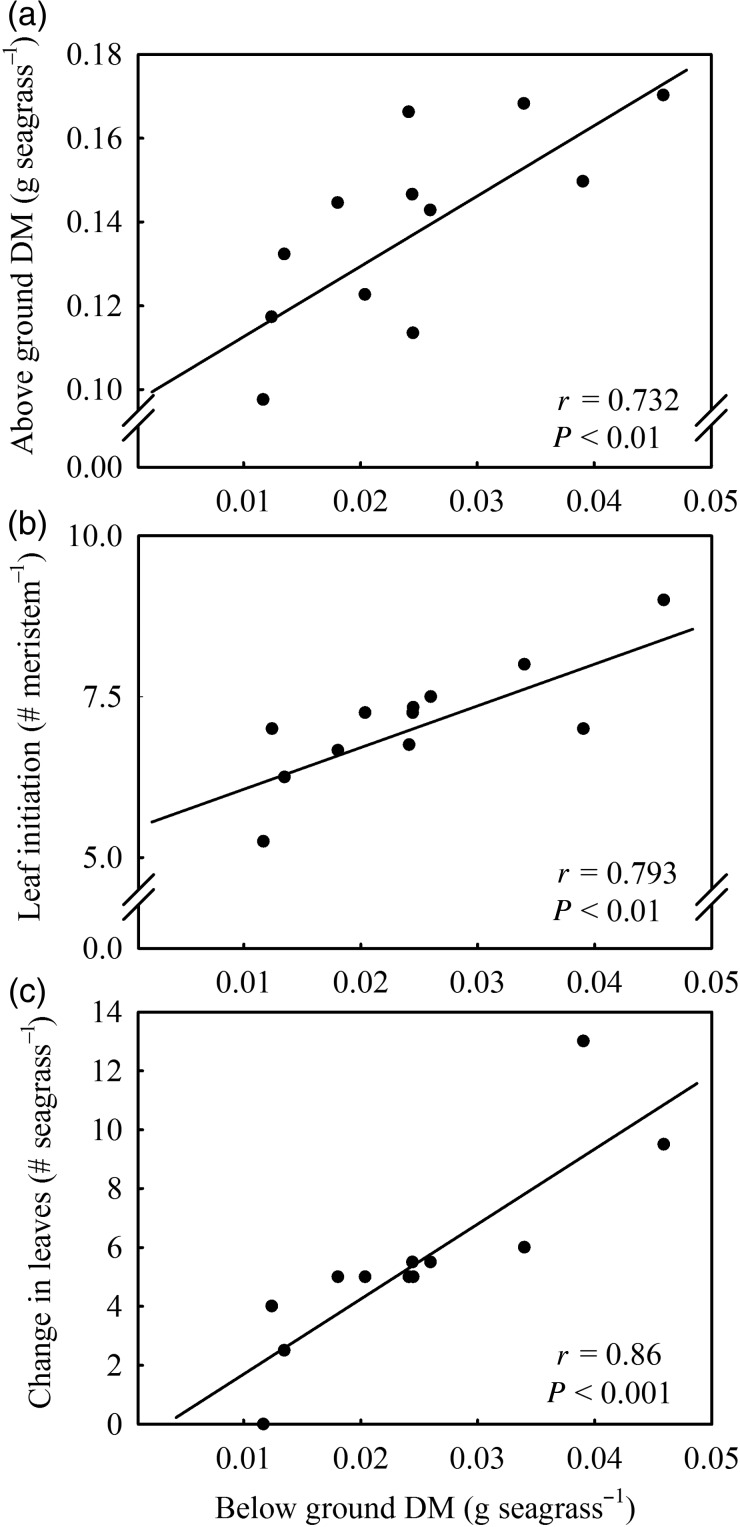
Correlations between below-ground biomass (expressed as DM per seagrass) and above-ground growth parameters (expressed as DM biomass per seagrass, leaf initiation per meristem and change in leaves per seagrass) for *A. antarctica*. Data points are the average in each microcosm (*n* = 6).

Growth of juvenile seagrass was greater at H[CO_2_], with total biomass (*F*_1,10_ = 4.26, *P* = 0.050), below-ground biomass (*F*_1,10_ = 5.42, *P* = 0.043) and change in leaf number (*F*_1,10_ = 5.40, *P* = 0.047) significantly higher than for L[CO_2_] grown plants. However, there was no significant difference in above-ground biomass (*F*_1,10_ = 2.79, *P* = 0.139) or leaf initiation from each individual meristem (*F*_1,10_ = 3.96, *P* = 0.087; Fig. 3a–e). Significant correlations between below-ground biomass and each of the above-ground growth parameters suggested that interdependent positive relationships existed between above- and below-ground growth (Fig. 4a–c).

## Discussion

The three seagrass species tested were sensitive to the inhibition of HCO_3_^−^-uptake mechanisms, indicating that at or under contemporary CO_2_ concentrations they are reliant on energetically costly C_i_ acquisition. Seagrasses are commonly known to use these HCO_3_^−^ pathways for photosynthesis (Beer *et al.*, 1977; Millhouse and Strother, 1986; James and Larkum, 1996; Beardall, *et al.*, 1998; Hellblom *et al.*, 2001); however, we have a limited understanding of how they regulate these mechanisms when faced with environmental variation (Larkum, *et al.*, 2006) and any implications of this for long-term growth. Any increase in the proportion of direct CO_2_ uptake relative to energetically costly HCO_3_^−^ acquisition could benefit their carbon balance and thus growth rate (Beardall and Giordano, 2002; Raven *et al.*, 2011; Koch *et al.*, 2013). In accordance with such predictions, we found that increased photosynthesis and growth of *A. antarctica* was accompanied by lower ETR-to-O_2_ ratios, indicating that not only growth but also photosynthetic efficiency could increase at forecasted CO_2_ concentrations.

Greater availability of CO_2_ increased the ETR_max_ in *A. antarctica*, as has been reported in other seagrass species (Jiang *et al.*, 2010; Alexandre *et al*., 2012). However, changes in ETR at forecasted CO_2_ concentrations were small relative to increases in light-saturated photosynthesis measured by O_2_ evolution (i.e. 1.4-fold for ETR vs. 2.1-fold for O_2_ evolution). This difference translated to lower ETR-to-O_2_ ratios calculated for H[CO_2_] plants when compared with L[CO_2_] plants (i.e. 8.08 vs. 9.51 mol electrons (mol O_2_)^−1^, respectively). This molar ratio is also commonly referred to in its inverse form, which would equate to 0.105 and 0.124 mol O_2_ (mol electrons)^−1^ for L[CO_2_] and H[CO_2_] plants, respectively. While these values differ from the theoretical maximum of 0.25 mol O_2_ (mol electrons)^–1^ that is based on the minimal number of electrons needed to produce a given amount of O_2_ during photosynthesis (i.e. 4 mol electrons (mol O_2_)^−1^; Walker, 1987), recorded values for different seagrass species are known to vary widely (Beer *et al.*, 1998; Silva *et al.*, 2009). Given that ETR-to-O_2_ ratios were measured at relatively high light intensities, it is likely that processes such as photorespiration contributed to these high ETR-to-O_2_ ratios. Photorespiration increases in conditions of low C_i_ availability, where plants fix O_2_ rather than CO_2_ (Black *et al*. 1976; Buapet *et al*. 2013). It is well known that the usually linear relationship between ETR and O_2_ evolution can deteriorate at high light intensities, at which photorespiration or other processes that can act as sinks for electrons increase (Beer *et al.*, 1998; Carr and Bjork, 2003; Silva and Santos 2004).

Photorespiration may also have contributed to the treatment differences that we observed between ETR-to-O_2_ ratios for L[CO_2_] and H[CO_2_] plants. Importantly, the finding that L[CO_2_] plants transported more electrons per mole of O_2_ evolved than H[CO_2_] plants could indicate greater photorespiration where plants were CO_2_ limited. In such circumstances, photorespiration is likely to have acted as a greater sink for electrons measured using fluorescence in L[CO_2_] plants. Thus, O_2_ evolution measurements may provide a better estimate of changing photosynthetic efficiency than ETR at higher irradiances, because photorespiration in CO_2_-limited individuals remains undetected by fluorescence measurements. However, photorespiration is generally low in seagrasses with effective carbon concentrating mechanisms that maintain high concentrations of CO_2_ around rubisco (Beer, 1989; Touchette and Burkholder, 2000). It is also possible that greater ETR-to-O_2_ ratios at low CO_2_ might be consistent with a greater proportion of electron flow supporting C_i_-uptake mechanisms. However, this may be of greater consequence at lower irradiances, where plants are forced to partition limited energetic resources between photosynthesis and CO_2_ acquisition.

Despite greater photosynthesis and growth at forecasted CO_2_ concentrations, there was limited evidence to suggest any down-regulation of HCO_3_^−^-uptake mechanisms in *A. antarctica* grown at H[CO_2_], because when seagrasses were switched to L[CO_2_] to measure photosynthesis, there was no effect of CO_2_ growth conditions. This suggests that, on transition to a different CO_2_ concentration, the photosynthetic rate was determined by changing dissolved CO_2_, rather than by different affinities for HCO_3_^−^ acquired during the 3 month growth period. If H[CO_2_]-grown plants were no longer able to acquire CO_2_ from HCO_3_^−^, much lower O_2_ production would have been expected when they were switched back to L[CO_2_]. Shorter-term studies have likewise found that the relative level of HCO_3_^−^ uptake by seagrass is often maintained across pH gradients (James and Larkum, 1996; Campbell and Fourqurean, 2013b). Thus, it appears that changes in growth and photosynthesis may have occurred based primarily on greater passive uptake of CO_2_, not on down-regulation of HCO_3_^−^-uptake mechanisms. Alternatively, down-regulation may have been masked if plants rapidly modified HCO_3_^−^-acquisition rates during the brief reciprocal CO_2_ transitions, although this appears unlikely. While rapid re-establishment (2–5 min) of HCO_3_^−^ acquisition can occur following dark-to-light transitions (Carr and Axelsson, 2008), the time for activation and deactivation for HCO_3_^−^ mechanisms with CO_2_ transitions would appear to be much greater. Studies of lower order producers (i.e. cyanobacteria and eukaryotic algae) and freshwater angiosperms show hours to days for full re-activation and down-regulation following CO_2_ transitions (Spalding and Ogren, 1982; Falk and Palmqvist, 1992; Matsuda and Colman, 1995; Maberly and Madsen, 2002).

The greater increase in below-ground biomass at H[CO_2_] suggests that *A. antarctica* may preferentially allocate resources to roots and rhizomes for growth and energy storage. These findings are consistent with studies of natural and *in situ* CO_2_ enrichment that indicate disproportionate accumulation of below-ground biomass in other seagrasses, relative to above-ground tissue (Hall-Spencer *et al.*, 2008; Russell *et al.*, 2013; Campbell and Fourqurean, 2013a). However, the significant correlation between below-ground biomass and the three above-ground growth parameters could suggest that the former simply responds more rapidly to CO_2_ enrichment, and this could later translate to changes in above-ground parameters. This translation to greater above-ground biomass would appear possible given that seagrass at H[CO_2_] also showed a greater increase in leaf number; nonetheless, with potentially greater carbon fixation per leaf area at enriched CO_2_ levels, there may be limited need for large increases in above-ground biomass.

The increase in below-ground biomass could offer seagrasses greater resistance to environmental perturbations in an era when anthropogenic influences have been reported to be almost exclusively negative (Orth *et al.*, 2006). Greater below-ground energetic resources could sustain juveniles during periods of physiological stress, including reduced light availability (Burke *et al.*, 1996; Mackey *et al.*, 2007; Ooi *et al.*, 2011), high temperature (Walker and Cambridge, 1995; Niu *et al.*, 2012; Koch *et al*., 2013) and grazing pressure (Hughes *et al.*, 2004). Preferential allocation to below-ground resources that are protected from herbivores might be particularly beneficial given that enriched CO_2_ conditions can result in seagrass that is more palatable to grazers owing to a reduction of phenolic deterrents (Arnold *et al.*, 2012). Likewise, faster establishment of below-ground structures could lower the vulnerability of seagrass to physical disturbances, such as wave action and bio-turbation/erosion (Townsend and Fonseca, 1998; Bastyan and Cambridge, 2008). An increase in recruitment success resulting from reproduction could be particularly beneficial, given that most seagrasses are heavily reliant on clonal growth for meadow expansion, with low rates of seed or seedling survival and natural recolonization (Kirkman and Kuo, 1990; Irving, 2013). Any prolonged increase in seagrass growth, however, will require other favourable conditions, such as adequate light, nutrients and facilitative interactions with other biota (i.e. grazer activity and epiphyte abundance), all of which are known to limit seagrass biomass where CO_2_ is enriched (O. W. Burnell, unpublished data; Palacios and Zimmerman, 2007; Alexandre, *et al.*, 2012; Burnell *et al.*, 2013).

As historically high CO_2_ levels potentially aided the initial transition of angiosperms into the aquatic realm (Beer and Koch, 1996; Beardall, *et al.*, 1998), forecasted increases in CO_2_ could return such benefits, by increasing the availability of dissolved CO_2_. We found that *A. antarctica* has a contemporary reliance on HCO_3_^−^-uptake mechanisms, as well as greater photosynthesis and growth with prolonged acclimation to forecasted CO_2_ conditions. Importantly, any lasting increase in productivity and growth could enhance the host of ecosystem benefits provided by seagrass meadows.

## Supplementary material

Supplementary material is available at *Conservation Physiology* online.

## Funding

S.D.C. and B.D.R. were funded by an ARC grant and S.D.C. by an ARC Future Fellowship.

## Supplementary Material

Supplementary Data
