# Supplementary material for: Contemporary reliance on bicarbonate acquisition predicts increased growth of seagrass Amphibolis antarctica in a high-CO2 world
Source: Conserv Physiol. 2014 Nov 27;2(1):cou052. doi: 10.1093/conphys/cou052 (PMC4732469; doi:10.1093/conphys/cou052)
Supplement: Supplementary Data [file supp_2_1_cou052__index.html]

Supplementary Data 

# Contemporary reliance on bicarbonate acquisition predicts increased growth of seagrass *Amphibolis antarctica* in a high-CO2 world

## Supplementary Data

Supplementary Data

**Files in this Data Supplement:**

- Supplementary Data - Doc file
