# Supplementary material for: Contemporary reliance on bicarbonate acquisition predicts increased growth of seagrass Amphibolis antarctica in a high-CO2 world
Source: Conserv Physiol. 2014 Nov 27;2(1):cou052. doi: 10.1093/conphys/cou052 (PMC4732469; doi:10.1093/conphys/cou052)
Supplement: Supplementary Data [file supp_cou052_cou052supp.doc]

**Supplementary Data**

**Contemporary reliance on bicarbonate acquisition predicts increased growth of seagrass *Amphibolis antarctica* in a high CO_2_ world**

**Materials and Methods**

*Additional species C_i_ acquisition mechanisms*

Seagrass leaves were cut into 30 mm sections for both species. For *Amphibolis griffithii* species the third youngest leaf from each leaf head was used. For *Posidonia sinuosa* the section was cut from 10 cm above the leaf sheath. These leaf positions were chosen as they were consistently free of epiphytes. All other methods were identical to those stated in the primary manuscript.

**Table S1.** ANOVA for photosynthesis in a) *Amphibolis griffithii* and b) *Posidonia sinuosa* measured in seawater (control), TRIS, AZ, and TRIS + AZ. Pairwise comparison (*Amphibolis griffithii*) TRIS, where –AZ, control > TRIS, where + AZ control = TRIS. AZ, where –TRIS, control > AZ, where +TRIS, control > AZ.

| **Source** | **df** | **MS** | ***F*** | **p** |
| --- | --- | --- | --- | --- |
| **a) *Amphibolis griffithii*** |  |  |  |  |
| TRIS | 1 | 44234 | 28.17 | 0.001 |
| AZ | 1 | 58550 | 37.29 | 0.001 |
| **TRIS × AZ** | **1** | **12367** | **7.88** | **0.008** |
| Residual | 28 | 1570 |  |  |
| **b) *Posidonia sinuosa*** |  |  |  |  |
| TRIS | 1 | 465 | 0.30 | 0.581 |
| AZ | 1 | 5851 | 3.80 | 0.058 |
| TRIS **×** AZ | 1 | 568 | 0.37 | 0.573 |
| Residual | 28 | 1539 |  |  |

**Note:** No significant differences were found between respiration rates for any treatments therefore analyses are not presented.

**Figure S1.** Rates of photosynthesis and respiration in a) *Amphibolis griffithii* and b) *Posidonia sinuosa* measured in seawater (control), TRIS, AZ, and TRIS + AZ. Bars are mean ± SE (*n* = 8).

**Table S2.** Rates of photosynthesis and respiration per gram of fresh mass (gfm) in a) *Amphibolis antarctica* (see Figure 1), b) *Amphibolis griffithii* (see Figure S1a) and c) *Posidonia sinuosa* (see Figure S1b) measured in seawater (control), TRIS, AZ, and TRIS + AZ. Rates of photosynthesis and respiration per gram of fresh mass in d) *Amphibolis antarctica* reciprocally switched between L[CO_2_] and H[CO_2_] treatments (see Figure 2b).

|  | | **Photosynthesis (µmol O_2_ gfm^-1^ min^-1^)** | | | **Respiration (µmol O_2_ gfm^-1^ min^-1^)** | |
| --- | --- | --- | --- | --- | --- | --- |
| **a) *Amphibolis antarctica*** | | | | | | |
| Control | | 0.171 | ± 0.020 | -0.039 | | ± 0.013 |
| TRIS | | 0.106 | ± 0.021 | -0.021 | | ± 0.013 |
| AZ | | 0.126 | ± 0.025 | -0.037 | | ± 0.009 |
| TRIS + AZ | | 0.097 | ± 0.018 | -0.032 | | ± 0.013 |
| **b) *Amphibolis griffithii*** | | | | | | |
| Control | | 0.263 | ± 0.014 | -0.022 | | ± 0.009 |
| TRIS | | 0.168 | ± 0.011 | -0.027 | | ± 0.011 |
| AZ | | 0.144 | ± 0.011 | -0.030 | | ± 0.009 |
| TRIS + AZ | | 0.112 | ± 0.012 | -0.019 | | ± 0.008 |
| **c) *Posidonia sinuosa*** | | | | | | |
| Control | | 0.167 | ± 0.024 | -0.036 | | ± 0.011 |
| TRIS | | 0.168 | ± 0.020 | -0.040 | | ± 0.012 |
| AZ | | 0.128 | ± 0.017 | -0.037 | | ± 0.010 |
| TRIS + AZ | | 0.116 | ± 0.012 | -0.042 | | ± 0.015 |
| **d) *Amphibolis antarctica*** | | | | | | |
| ML[CO_2_] + GL[CO_2_] | 0.107 | | ± 0.029 | -0.035 | | ± 0.008 |
| ML[CO_2_] + GH[CO_2_] | 0.092 | | ± 0.024 | -0.026 | | ± 0.009 |
| MH[CO_2_] + GL[CO_2_] | 0.256 | | ± 0.048 | -0.027 | | ± 0.016 |
| MH[CO_2_] + GH[CO_2_] | 0.253 | | ± 0.044 | -0.041 | | ± 0.009 |
